# Hormone-modulated transcription factors orchestrating the root nodule symbiosis

**DOI:** 10.3389/fpls.2026.1811506

**Published:** 2026-04-15

**Authors:** Cristina Kirolinko, Milagros Yacullo, Flavio Blanco, María Eugenia Zanetti

**Affiliations:** Instituto de Biotecnología y Biología Molecular, Facultad de Ciencias Exactas, Universidad Nacional de La Plata, Centro Científico y Tecnológico-La Plata, Consejo Nacional de Investigaciones Científicas, La Plata, Argentina

**Keywords:** auxin, cytokinin, ethylene, gibberellins, nitrogen fixation, nodulation, symbiosis, transcription factors

## Abstract

Transcription factors play essential roles modulating gene expression during plant development and the adaptation to environmental cues through the control of morphogenetic programs. In the root nodule symbiosis between legumes and rhizobia, two coordinated morphogenetic programs are activated by the perception of bacterial signals: the organogenesis of the nodule, a lateral root organ specialized in nitrogen fixation, and the infection process that allows the bacteria to colonize the nodule. These programs are influenced by the action of phytohormones, mainly auxin, cytokinin, ethylene, gibberellin, and brassinosteroid, which act modulating the activity of different families of transcription factors. In the past years, significant advancements have been made in understanding how transcription factors of the NIN (Nodule Inception), GRAS (GIBBERELLIN-ACID INSENSITIVE (GAI), REPRESSOR of GA1 (RGA), and SCARECROW (SCR)), ERF (Ethylene Response Factor), ARF (Auxin Response Factor), LBD (Lateral Organ Boundaries Domain), and SHI/STY (SHORT INTERNODES/STYLISH) families function at different developmental stages of bacterial infection and nodule formation and differentiation. Here, we review recent advances of this hormonal-mediated modulation of transcription factors with key roles in the root nodule symbiosis and their evolutionary origin from other developmental programs, as well as their post-transcriptional regulation by small RNAs. We also provide a perspective on how epigenomic approaches can shed light on how these transcription factors influence chromatin remodeling at *loci* containing key symbiotic genes.

## Introduction

Plant hormones play crucial roles in integrating developmental programs with environmental signals to control gene expression. Each of them has distinct receptors and signaling pathways that ultimately activate transcription factors, which act on DNA to turn specific genes on or off. In addition, cross-regulation between hormone signaling pathways might occur through transcription factors shared between different pathways and/or by the regulation of shared downstream genes activated by independent transcription factors (Nemhauser et al., 2006; Yin et al., 2023). Intensive investigation over the past 30 years has revealed how different phytohormones modulate the root nodule symbiosis between legume plants and rhizobia (Lin et al., 2020). Here, we will summarize the current knowledge of the bidirectional crosstalk between hormones and transcription factors, i.e., how hormone signaling influences the activity of transcription factors, and how transcription factors control the expression of hormone biosynthetic and responsive genes, during the root nodule symbiosis (Lin et al., 2020; Yin et al., 2023). First, we will provide a brief introduction to the root nodule symbiosis and describe how different hormones impact on the development of nitrogen fixing nodules. Then, we will focus on regulatory and evolutionary aspects of each of the different transcription factor families modulated by hormones, as well as the interaction between them, which ultimately orchestrate the morphogenetic programs of rhizobial infection and nodule organogenesis. We will pay special attention to those transcription factors modulated by cytokinin, gibberellin, auxin, ethylene, and brassinosteroids for which genetic and molecular mechanism has been uncovered. Finally, we provide a perspective for the advancement in identifying *cis*-regulatory elements in key symbiotic genes and emphasize how epigenomic approaches can assess their accessibility for the binding of hormone-modulated transcription factors during the root nodule symbiosis.

## The morphogenetic programs of infection and nodule organogenesis

Plants within the nitrogen fixation clade (NFC), which includes the orders *Fabales*, *Fagales*, *Cucurbitales* and *Rosales*, acquired the capacity to establish an intimate symbiotic interaction with diazotrophic bacteria to obtain reduced forms of nitrogen in exchange for photosynthetic compounds and a low oxygen microenvironment suitable for nitrogen fixation (Soltis et al., 1995; Werner et al., 2014). Legumes (*Fabales*) and plants from the genus *Parasponia* (*Rosales*) associate with gram negative bacteria known as rhizobia, whereas actinorhizal plants form nodules with Gram-positive bacteria from the genus *Frankia*, resulting in the development of a new organ specialized in nitrogen fixation, the nodule, where the bacteria will lodge (Kundu et al., 2025). Two independent but highly coordinated genetic programs are executed in nodulating plants, the bacterial infection and the nodule organogenesis.

**Figure 1:**
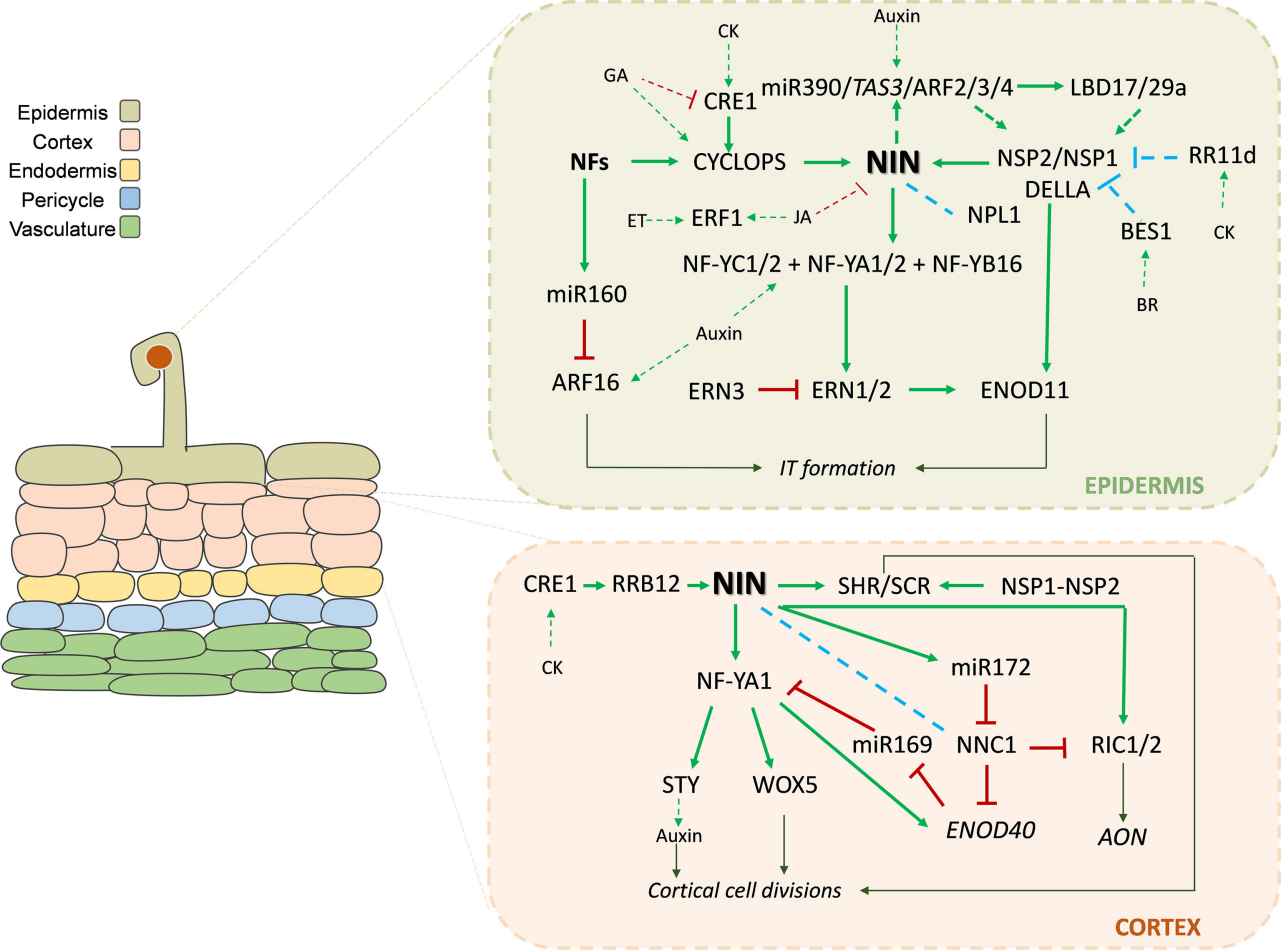
Hormone-modulated transcriptional networks controlling rhizobial infection in the epidermis and cell divisions in the cortex. Schematic representation of the transcriptional and hormonal regulatory networks controlling early rhizobial infection and cortical cell divisions in legume roots. In the epidermis, perception of rhizobial signals activates the CSSP, leading to the activation of transcription factors such as CYCLOPS, NIN, NSP1/NSP2, and ERN1/2, which promote infection thread (IT) initiation and progression. Brassinosteroid (BR) act through BES1 to inhibit NSP1/NSP2 heterodimer formation and inactivate NSP1., whereas cytokinin (CK) activates RRB11d to inactivate NSP1 transcriptional activity on NIN in the epidermis. In the cortex, CK perception through the receptor CRE1 activates RRB12 mediated expression of NIN. In turn, NIN mediates activation of transcriptional programs in the cortex, including NF-Y complexes (NF-YA1/2, NF-YB16, NF-YC1/2), LBD transcription factors, and the SHR/SCR module, promoting and coordinating cortical cell divisions with infection. Auxin signaling components, including ARF16 and the miR160 module, act locally to modulate epidermal infection responses. In parallel, crosstalk between auxin, CK, gibberellic acid (GA), and jasmonic acid (JA) signaling pathways ensure spatial and temporal control of IT formation and progression toward the cortex. Solid green arrow lines indicate activation; red blunt-ending lines indicate inhibition; green dotted arrow lines indicate indirect or hormonal responses; blue dotted lines indicate interaction between transcription factors, blue doted blunt-ended lines indicate inhibition by interaction between transcription factors.

Infection by rhizobia occurs mainly by two alternative mechanisms. Intercellular infection, where bacteria enter between epidermal plant cells, is considered the most ancient mechanism, whereas intracellular infection is a more sophisticated mechanism that involves the formation of tubular structures called infection threads (ITs). In several agronomically important legumes, as well as in the two model legumes *Medicago truncatula* and *Lotus japonicus*, infection by rhizobia occurs by the intracellular mechanism. This infection mechanism is initiated by the attachment of the bacteria to the growing root hairs, which curl to entrap the bacteria into a structure called the infection pocket (Murray, 2011) (**Figure 1**). The subsequent degradation of the plant cell wall and the plasma membrane initiate the formation of the IT that grows inward toward the cortical cells, where they ramified (Fournier et al., 2015; Murray, 2011). Bacteria are released from the ITs into cortical cells and surrounded by a plant-derived membrane forming organelle-like structures known as symbiosomes, where bacteria differentiate to bacteroids and begin to actively fix atmospheric nitrogen (Jhu and Oldroyd, 2023).

**Figure 2:**
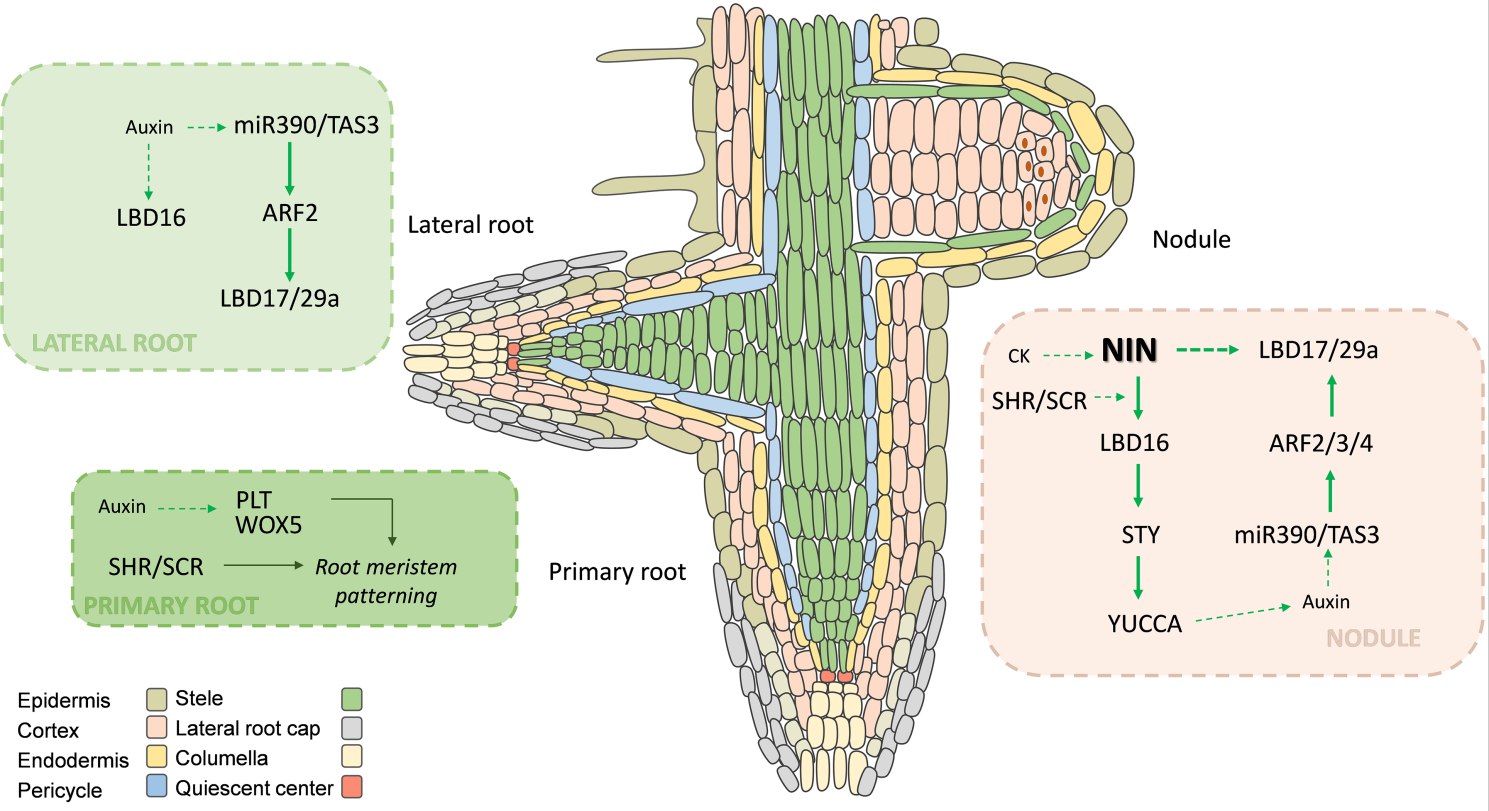
Recruitment of root and lateral root developmental programs during nodule organogenesis. Integration of hormone signaling and transcriptional regulators during root and lateral root development and nodule organogenesis. Cytokinin (CK) signaling via CRE1 and the central regulator NIN activates transcriptional programs shared with lateral root development, including the miR390/TAS3/ARF2 module and LBD transcription factors such as LBD16 and LBD17/29a. These factors promote cortical cell divisions required for nodule primordium formation. Activation of STY transcription factors and YUCCA genes enhances local auxin biosynthesis, reinforcing auxin maxima in dividing cortical cells. The coordinated action of NF-Y complexes, ARFs, and LBDs illustrates the evolutionary recruitment of lateral root regulatory modules to drive nodule initiation. In addition, SCR and SHR have been recruited from the root radial development program to act in the activation of cortical cell divisions mediated by CK, NIN and LBD16 during nodule development. Solid green arrow lines indicate activation; dotted green arrow lines indicate indirect or hormonal response.

**Figure 3:**
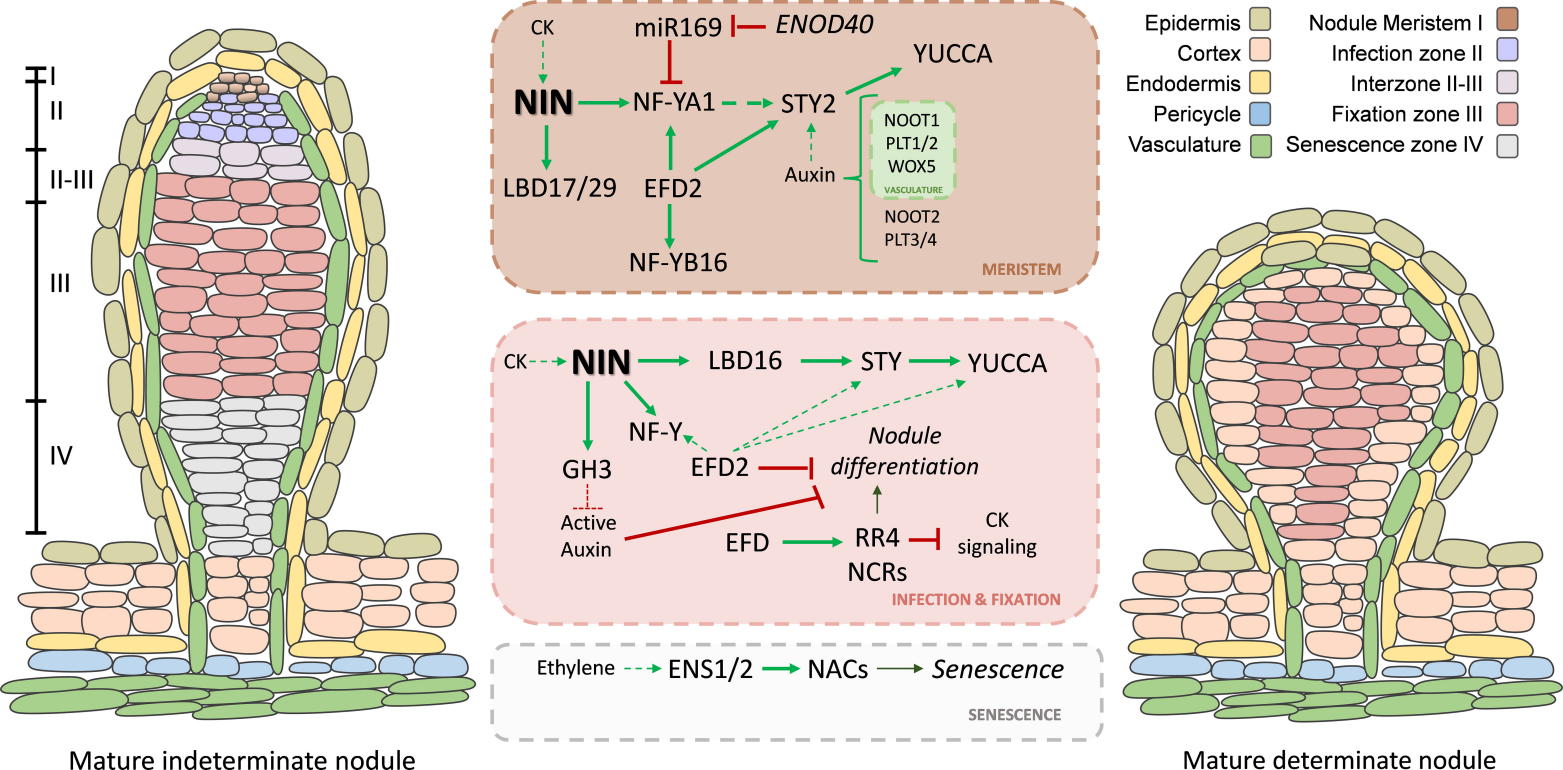
Transcriptional and hormonal regulation in mature indeterminate and determinate nodules. Model summarizing the transcriptional networks controlling meristem maintenance, infection, fixation, and senescence zones in mature indeterminate nodules (left). In the nodule meristem, NF-YA1 expression is spatially regulated by miR169 and the endogenous mimicry RNA curiva, while WOX5 and PLETHORA (PLT) transcription factors contribute to meristem identity. LBD17/29, STY2, and YUCCA genes promote auxin biosynthesis in the meristem and uninfected cells of the fixation zone. NOOT1/NOOT2 contribute to the maintenance of nodule identity and vascular organization by modulating auxin–cytokinin balance. In the infection and fixation zones, NIN, NF-Ys, EFDs, and the cytokinin-responsive regulator RR4 coordinate bacterial accommodation, bacteroid differentiation, and nitrogen fixation. In older nodules, ethylene signaling pathway modulates nodule senescence. NIN-mediated activation of GH3 reduces levels of active auxin, contributing to disappearance of the infection zone in determinate nodules (right). In this type of nodules, senescence is regulated by the ERF/AP2 transcription factors ENS1 and ENS2, which directly activate NAC transcription factors. Solid green arrow lines indicate activation; red blunt-ending lines indicate inhibition; dotted green arrow lines indicate indirect or hormonal response.

Nodule organogenesis begins with the activation of cell divisions in the inner root cell layers (**Figures 1**, **2**). The cell layers where cell division is activated differ in the two types of symbiotic nodules formed in legume plants: determinate and indeterminate types. In legumes that form the determinate type of nodules, initial cell divisions are activated in the pericycle and in the middle or outer cortex (Hirsch, 1992), whereas in legumes with the indeterminate type of nodules, cell divisions are activated in the pericycle, endodermis and the inner cortical cells (Timmers et al., 1999; Xiao et al., 2014). A hallmark of indeterminate nodules is the presence of a persistent meristem that continuously adds cells during the life span of the nodule, resulting in distinct differentiated zones: the meristematic zone, the infection zone, the interzone, the fixation zone and, in older nodules, the senescence zone (Jhu and Oldroyd, 2023) (**Figure 3**). Determinate nodules, which lack a persistent meristem, have been recently shown to contain distinct zones, an undifferentiated pre-infection and infection zones and a differentiated nitrogen fixation zone (Tu et al., 2024). Independently of the type of nodules, accumulating evidence indicates that components of the other root developmental programs, mainly transcription factors, have been recruited and neofunctionalized to participate in the activation of cell division that give rise to nodule formation (Soyano et al., 2021; Schiessl and Jhu, 2025).

The morphogenetic programs of infection and nodule organogenesis are activated by the perception of lipo-chito-oligosaccharide signal molecules produced by rhizobia, the Nod Factors (NFs). NFs are recognized by receptor-like kinases that contain extracellular LysM domains (Amor et al., 2003; Radutoiu et al., 2003; Smit et al., 2007). This recognition event activates a signaling pathway referred to as the Common Symbiosis Signaling Pathway (CSSP), since it is present not only in the root nodule symbiosis, but also in the most ancient arbuscular mycorrhizal symbiosis (Oldroyd and Downie, 2008; Kundu et al., 2025). The CSSP includes components of signal transduction such as receptor like kinases, ion channels, and oscillations in calcium concentration referred to as calcium spiking, which are decoded by a calcium calmodulin dependent protein kinase (CCaMK) that interacts with a transcription factor called CYCLOPS activating a cascade of downstream transcription factors that are specific for the formation of functional nitrogen fixing nodules (Soyano et al., 2021), which are modulated by different plant hormones as will be described in detail in the following sections.

## Hormonal control of morphogenetic programs in the root nodule symbiosis

Plant hormones orchestrate the morphogenetic programs required for the establishment of nitrogen fixing nodules, including infection, nodule organogenesis and cell differentiation during nodule development, by intersecting the nodulation signaling pathway through modulation of transcription factors (Lin et al., 2020). In this section, we will present advancements describing the role of plant phytochromes that influence bacterial infection and nodule organogenesis.

Several lines of evidence support the positive role for cytokinin in the formation of nitrogen fixing nodules (Gamas et al., 2017). Application of cytokinin to roots was shown to induce nodule-like structures in several species (Plet et al., 2011; Held et al., 2014; Gauthier-Coles et al., 2018) and genes involved in cytokinin biosynthesis are expressed in the cortex after inoculation with rhizobia in *L. japonicus* and *M. truncatula* (Mortier et al., 2014; Reid et al., 2017). More recently, it has been evidenced that cytokinin response to rhizobia exhibited a periodic oscillation pattern that influences the size of the root zone susceptible to rhizobial infection and the progress of nodulation (Soyano et al., 2024). Overexpression or gain of function mutants of the cytokinin receptor *LOTUS HISTIDINE KINASE (LHK1*) in *L. japonicus* activates cortical cell divisions, resulting in spontaneous nodule formation (Tirichine et al., 2007; Liu et al., 2018), whereas loss of function mutants of *LHK1* in *L. japonicus* or of the *Cytokinin Receptor 1* in *M. truncatula* (*MtCRE1*) showed reduced nodulation as compared to wild type plants when they were inoculated with rhizobial strains that exhibited the intracellular infection mechanism (Murray et al., 2007; Plet et al., 2011). Interestingly, cytokinin signaling and response, as well as the expression of cytokinin biosynthetic genes, appears to be differently regulated depending on the infection mechanism, which is supported by evidence that showed that *L. japonicus lhk1* mutant formed no nodules upon inoculation with a strain with the intercellular infection mechanisms (Montiel et al., 2021).

Auxin signaling influences both the initiation of ITs and the nodule organogenesis (Breakspear et al., 2014; Xiao et al., 2014, Xiao et al., 2025). Auxin appears to act as positive modulator of IT formation and auxin responses increased in root hairs infected with rhizobia or treated with NFs in both *M. truncatula* and *L. japonicus* (Breakspear et al., 2014; Nadzieja et al., 2018). The role of auxin in nodulation was initially established by the use of auxin transport inhibitors, which trigger nodule formation (Hirsch et al., 1989). It has been suggested that flavonoid production in response to rhizobia affects auxin transport, producing local changes of auxin/cytokinin levels that trigger cell-cycle reactivation (Mathesius, 2001). Auxin signaling and response coincide with the site of initial cell division that leads to the formation of both determinate and indeterminate nodules (Takanashi et al., 2011; Kohlen et al., 2018; Xiao et al., 2025). In addition, activation of auxin biosynthesis seems to be a function of cytokinin signaling during nodule formation (Schiessl et al., 2019). All this evidence points to a local modulation of cytokinin/auxin levels to produce the reinitiation of the cell cycle in the endodermis, pericycle and cortex to give origin to the nodule.

Gibberellic acid (GA) has been shown to exert contrasting effects in nodule organogenesis and rhizobial infection (Velandia et al., 2026). Cell layer-specificity seems to govern the GA-mediated control of these two processes during nodulation. Production of GA in the endodermis promotes nodule organogenesis, and also lateral root formation; however, GA production in the epidermis appears to suppress bacterial infection and restrict the progression of ITs toward the cortical cells in pea (*Pisum sativum*) roots (Velandia et al., 2024). In addition, local GA accumulation at the site of nodule primordium formation, particularly in the cortical cell layers, is required for nodule organogenesis and development in *M. truncatula* (Drapek et al., 2024).

With the exception of the semiaquatic legume *Sesbania rostrata*, in which ethylene promotes nodulation (D'Haeze et al., 2003), ethylene has been shown to act as a negative modulator of rhizobia infection and nodule organogenesis in several legume species (Lee and Larue, 1992; Wood, 2001). In *M. truncatula*, the ethylene insensitive mutant *sickle* (*skl*), which is affected in the *EIN2* (*Ethylene Insensitive 2*) gene, exhibited hyperinfection and excessive nodule primordia that do not develop into mature nodules (Penmetsa and Cook, 1997; Prayitno et al., 2006). Similarly, *L. japonicus ein2a ein2b* double mutants exhibited hyperinfection and hypernodulation; however, they were impaired in nitrogen fixation (Reid et al., 2018). In addition, direct and indirect exogenous application of ethylene or the inhibition of ethylene biosynthesis revealed that this hormone interferes with all the major early responses to rhizobia, i.e., root hair deformation and initiation and progression of ITs, as well as responses to NF, including calcium spiking (Oldroyd et al., 2001).

Brassinosteroid appears to have positive and negative effects in infection and nodule organogenesis depending on the legume species. In pea plants, brassinosteroids suppress rhizobial infection by inhibiting active auxin accumulation in root hairs, root hair deformation and IT formation, whereas they have a positive effect on the initiation of nodule organogenesis in the cortex (McGuiness et al., 2020). Pea plants mutated in genes involved in brassinosteroid biosynthesis or in the gene encoding the brassinosteroid receptor *LKA* exhibited reduced number of nodules (Nomura et al., 2003; Ferguson et al., 2005). Similarly, *M. truncatula* plants mutated in the brassinosteroid receptor *MtBRI1* exhibited reduced nodulation (Cheng et al., 2017). However, in soybean brassinosteroid signaling appears to inhibit nodulation by suppressing the NF signaling pathway (Chen et al., 2023). Overexpression of GmBRI1b receptor or a downstream transcription factor reduced nodulation (Chen et al., 2023), revealing opposite functions of brassinosteroid signaling in legume species forming indeterminate or determinate type of nodules.

Other hormones, such as abscisic acid, jasmonic acid (JA), and strigolactones regulate positively or negatively different aspects of nodule symbiosis. The role of hormones in nodule organogenesis and bacterial infection has been recently reviewed in detail (Lin et al., 2020; Velandia et al., 2022). In the following section, we will focus on how different families of hormone-modulated transcription factors participate in each of the steps that give origin to a functional nitrogen-fixing nodule in legume plants.

## Hormone-modulated transcription factors with roles in the root nodule symbiosis

### Nodule inception, the central regulator of infection and nodule organogenesis, integrates multiple hormone signaling pathways

Nodule Inception (NIN) is a transcription factor originally identified in *L. japonicus* (Schauser et al., 1999) that gave the name to a family of transcription factors referred to as NIN-like proteins (NLPs), which are characterized by the presence of a RWP-RK DNA binding domain and a Phox and Bem1 (PB1) domain that facilitates protein-protein interactions (Sámano et al., 2024). NIN functions as a central regulator coordinating the two morphogenetic programs required for the formation of functional nitrogen-fixing nodules, bacterial infection and organogenesis (Schauser et al., 1999; Borisov et al., 2003; Marsh et al., 2007; Soyano et al., 2013, Soyano et al., 2014; Clavijo et al., 2015; Lin et al., 2018; Liu et al., 2019; Bu et al., 2020; Feng et al., 2021). It is also at the center of the evolutionary speculations around the origin of nodulation. Recent works suggest that the acquisition of the symbiotic ability was a single genetic event in a common ancestor of the NFC that was later lost on many occasions as a consequence of mutations that disrupted *NIN* function (Griesmann et al., 2018; van Velzen et al., 2018; Shen et al., 2020). The fact that NLPs have been shown to act in non-legume species such as tomato and Arabidopsis functioning in the nitrate signaling pathway (Konishi and Yanagisawa, 2013; Liu et al., 2021) and that NIN interact with NLP1 to mediate nitrate inhibition of nodulation (Lin et al., 2018), suggests that NIN could have been evolutionary recruited from plant responses to nitrogen (Liu and Bisseling, 2020; Soyano et al., 2021). Recent research showed that the N-terminal region of NLP1, which is essential for nitrate sensing, is conserved in NPL members but contains mutation or deletions in the NIN protein, highlighting the importance of this N-terminal region for proper function of both NLP and NIN in nitrate signaling and nodulation (Wang et al., 2025).

The key to *NIN* neofunctionalization seems to be located on its promoter, which drives the nodule-specific expression in all legumes studied. As previously mentioned, triggering of CSSP by LysM receptors results in the activation of CYCLOPS, a coiled-coil transcription factor that binds a CYCLOPS-responsive *cis*-element present in the *NIN* promoter of legumes to control infection in the epidermal cells (Singh et al., 2014; Liu et al., 2019; Akamatsu et al., 2022; Cathebras et al., 2026). It has been reported that GA activates *NIN* expression through this CYCLOPS-responsive *cis*-element (Akamatsu et al., 2022). In turn, NIN activates GA biosynthetic genes, generating a signaling loop that controls rhizobial infection in the context of the autoregulation of nodulation (AON), a regulatory system that balances the number of nodules formed with the nitrogen requirements and energy availability (Gao et al., 2023) (**Figure 1**). NIN has been shown to control rhizobium infection modulating the expression of symbiotic genes such as *Nodule Pectate Lyase* (*NPL), RHIZOBIUM-DIRECTED POLAR GROWTH* (*RPG*) and *SCAR-Nodulation* (*SCARN*) (Arrighi et al., 2006; Xie et al., 2012; Qiu et al., 2015; Lace et al., 2023; Li et al., 2023).

Expression of *NIN* in inner cortical cells and the pericycle is controlled by another *cis*-regulatory element named CE (cytokinin response element-containing region) present in nodulating legumes (Liu et al., 2019; Liu and Bisseling, 2020; Zhang et al., 2024). This remote element, located 18 kb upstream of the translation start codon, contains ten putative cytokinin B-type Response Regulator (RR) binding sites and mediates *NIN* activation via the cytokinin receptor *LHK1*, triggering subsequent cell divisions in the pericycle and cortex that give origin to the nodule primordia in *L. japonicus* (Liu et al., 2019). B-type RR are considered authentic histidine phosphotransferase that transmit hormonal signaling by activating cytokinin-responsive transcription factors in the nuclei (Kieber and Schaller, 2018). It has been recently shown that the B-type RR of *L. japonicus* LjRRB12 positively modulates nodule formation by binding to the CE in the *LjNIN* promoter, providing a link between cytokinin signaling and transcriptional control of *NIN* (Cao et al., 2024). Additional evidence about the link between cytokinin and NIN expression came from the demonstration that gain of function mutants in *LHK1* (*snf2* and *snf5*) exhibited high *NIN* mRNA levels in the absence of rhizobia, which is correlated with the spontaneous formation of nodules in a *NIN*-dependent manner (Tirichine et al., 2007; Liu et al., 2018). In *M. truncatula*, NIN and MtCRE1 form a positive feedback loop that triggers nodule development (Vernié et al., 2015; Dong et al., 2021). Altogether, these results illustrate the central role of cytokinin in *NIN* expression to positively control nodule organogenesis (**Figure 1**).

NIN is also strongly connected with auxin-mediated gene regulation through transcription factors that are direct targets of NIN, including members of the Nuclear Factor Y (NF-Y) and Lateral Organ Boundaries Domain (LBDs) families (Schiessl et al., 2019; Soyano et al., 2019), as will be described in the following sections. In addition, GmNIN2 controls the expression of the *GRETCHEN HAGEN 3* (*GH3*) genes involved in the deactivation of auxin, reducing the levels of physiologically active auxin and facilitating cell differentiation and disappearance of the infection zone in soybean nodules (Tu et al., 2024). In the actinorhizal plant Datisca, the auxin phenylacetic acid induces *NIN1*, but in the presence of the synthetic cytokinin 6-Benzylaminopurine this induction is abolished, showing that hormonal regulation of *NIN* expression differs between legumes and actinorhizal plants (Salgado et al., 2025).

Modulation of *NIN* expression by other phytohormones is also possible and remains to be investigated. For instance, JA, a negative regulator of nodule and lateral root formation, has been shown to impair *NIN* induction in response to rhizobia in *L. japonicus* (Nakagawa and Kawaguchi, 2006). However, the underlying mechanism and the *cis*-regulatory elements within the *NIN* promoter responsible for this negative control have not been yet elucidated.

### GRAS transcription factors integrate hormonal responses to connect CSSP with the nod signaling pathway

GRAS comprises a plant-specific group of proteins named after the first three members of the family: GIBBERELLIC ACID INSENSITIVE (GAI), REPRESSOR of GAI (RGA) and SCARECROW (SCR). They have been associated with morphogenetic programs and hormonal control of environmental responses (Hirsch and Oldroyd, 2009; Khan et al., 2022). In different plant species, including legumes, GRAS proteins have been divided into different subfamilies according to phylogenetic analyses and the presence of functional motifs including the DELLA, HAM, LS, LISCL, NSP2, PAT1, SCR, SCL3, and SHR subfamilies (Tian et al., 2004; Lee et al., 2008; Battaglia et al., 2014).

The GRAS proteins NODULATION SIGNALING PATHWAY 1 (NSP1) and NSP2 (Kalo et al., 2005; Smit et al., 2005) form homo and heterocomplexes that bind to the Nodulation Response (NR) cis-regulatory element present in the promoter of early nodulation genes, including *MtENOD11* and *MtERN*, to activate their expression in response to rhizobia (Hirsch et al., 2009). The complex formed by NSP1 and NSP2 also directly binds the *NIN* promoter to control its expression very early during the symbiotic association, activating downstream transcriptional reprogramming (Hirsch et al., 2009) (**Figure 1**). The NSP1/NSP2 heterocomplex can also include other transcription factors, such as a MYB in *L. japonicus* or a GRAS in *Melilotus albus* (Xiao et al., 2020; Wang et al., 2025). More recently, it was shown that the brassinosteroid signaling negatively affects NF signaling and nodule formation through the transcription factor *bri1 (brassinosteroid insensitive)*-EMS-Suppressor 1 (GmBES1). GmBES1 was shown to interact with GmNSP1 and GmNSP2 in the nuclei of soybean root hairs to inhibit the formation of the GmNSP1/GmNSP2 heterocomplex and the DNA binding activity of GmNSP1, compromising the activation of the symbiotic genes *GmERN1* and *GmENOD93* (Chen et al., 2023). Interestingly, the B-type RR GmRR11d, which is induced by rhizobia and cytokinin in soybean, interacts with GmNSP1a to systemically inhibit nodulation by suppressing its transcriptional activity on *GmNIN1a* expression, providing a link between cytokinin signaling and these two transcription factors in the control of autoregulation of nodulation (Chen et al., 2022). Another GRAS protein, Required for Arbuscular Mycorrhization 1 (RAM1), interacts with NSP2 to induce specific responses associated with mycorrhization (Gobbato et al., 2012). This suggests that this family of transcription factors play critical roles in triggering the specific developmental programs of each symbiosis downstream of the CSSP. Interestingly, both *NSP1* and *NSP2* genes are required for strigolactone biosynthesis in *M. truncatula* and rice, and *nsp1/nsp2* exhibited reduces arbuscular mycorrhizal colonization (Liu et al., 2011). In addition. it has been shown that single *nsp2* mutants exhibited reduced biosynthesis of strigolactones, which are also required to promote infection thread formation during the root nodule symbiosis (McAdam et al., 2017).

DELLA proteins belong to a subfamily of GRAS that include non-canonical transcriptional regulators (Xue et al., 2022). In the root nodule symbiosis, DELLAs participate in the formation of the NSP1/NSP2 complex, whereas they have a broader participation in the CSSP by forming a complex with the CCaMK and the transcription factor CYCLOPS/IPD3 (Interacting protein with DMI3) (Jin et al., 2016) (**Figure 1**). *M. truncatula* roots mutated in *DELLA1* and *DELLA2* genes display lower transcript levels of *NSP1* and *NSP2*, indicating that DELLA proteins control directly or indirectly transcriptional activity of these genes (Floss et al., 2013). DELLA proteins integrate hormonal control with transcriptional regulation by forming complexes with DNA-binding factors. Binding of the receptor gibberellin-insensitive dwarf-1 (GID1) to GA triggers degradation of DELLA proteins via the proteasome (Davière and Achard, 2013). The negative role of GA and the positive role of DELLA proteins in rhizobial infection and NF-mediated signal transduction have been evidenced in *M. truncatula* using exogenous application of GA or a GA-antagonist (PAC) in combination with DELLA mutants (Fonouni-Farde et al., 2017). Similarly, loss of function mutants of two DELLA genes in pea showed that the effect of GA is mediated by DELLA proteins (Ferguson et al., 2011). In *M. truncatula*, the DELLA-mediated effect of GA involves a reduction of bioactive cytokinin, affecting the NF response mediated by MtCRE1 and NSP2 (Fonouni-Farde et al., 2017). The same authors showed that expression of a dominant active version of DELLA1 can trigger nodule-like structures and partially rescue the phenotype of the *cre1* mutant, indicating that DELLAs mediate GA signaling interplaying with the cytokinin-CRE1 pathway to control nodulation.

SCARECROW (SCR) -the first identified member of the GRAS family- and SHORTROOT (SHR) are involvedin the control of root radial patterning and root growth in *A. thaliana* (Helariutta et al., 2000). Expression of *SCR*is limited to the quiescent center and endodermis in non-legume plants, whereas *SCR* genes from different legume species are also expressed in cortical cells as a function of the cytokinin mediated activation of *NIN* (Dong et al., 2021) (**Figure 1**). Two elements present in the *M. truncatula* promoter of *SCR* were identified: the AT1-box and an enhancer, which are located close to each other in the promoters of *SCR* genes in legumes. Mutation of *SCR* genes or expression of an RNA interference (RNAi) targeting *MtSHR1* in a *Mtshr2* mutant background affected nodule organogenesis, showing that this module was evolutionary co-opted to control cortical cell divisions induced by rhizobia. Induction of *SCR* expression in *M. truncatula* requires MtSHR1/2, as well as NSP1/NSP2 and NIN (Dong et al., 2021) (**Figures 1**, **2**). As in the case of NIN, this example illustrates the importance of changes in gene expression caused by the acquisition of promoter *cis*-regulatory elements that produced neofunctionalization in the evolutionary origin of root nodule symbiosis.

### APETALA2/ethylene response factors act as positive and negative modulators of the root nodule symbiosis

Ethylene is perceived by a family of receptors localized in the endoplasmic reticulum (ER). In the absence of ethylene, the protein kinase constitutive triple response 1 (CTR1) inhibits an endoplasmic reticulum transmembrane protein called ethylene-insensitive 2 (EIN2), preventing downstream signaling. In the presence of ethylene, CTR1 is inactivated by the receptors, releasing the inhibition of EIN2, which is dephosphorylated and cleaved. The C-terminal of EIN2 is translocated to the nucleus, where it binds to and stabilizes the EIN3 and EIN3-like (EIL) transcription factors, which in turn activate expression of downstream APETALA2/Ethylene Response Factors (AP2/ERFs) to modulate the expression of ethylene responsive genes (Binder, 2020). The AP2/ERF is a superfamily of transcription factors characterized by the presence of at least one DNA binding domain of about 60 to 70 amino acids referred to as the AP2/ERF domain. The AP2/ERF superfamily has been divided into three subfamilies: AP2, ERF and RAV. Proteins of the AP2 subfamily contain two separated AP2/ERF domains, the ERF subfamily contains a single AP2/ERF domain, and the RAV subfamily contains a single AP2/ERF domain and an additional DNA-binding domain conserved in other plant-specific transcription factors, the B3 domain (Nakano et al., 2006). The ERF subfamily was further divided into 12 and 15 groups in Arabidopsis and rice, respectively (Nakano et al., 2006). Although ethylene seems to be a negative modulator of early signaling, infection by rhizobia and nodule organogenesis in several legume species (Oldroyd et al., 2001; Wood, 2001), individual members of the AP2/ERF superfamily have been shown to act either as positive or negative modulators of the different steps that lead to the formation of functional nitrogen fixing nodules in distinct legume species.

A seminal work describing the role of AP2/ERFs in the root nodule symbiosis reported that loss offunction mutations in the *M. truncatula ERF Required for Nodulation 1*(*MtERN1*) gene impair the initiation and progression of ITs and thus bacterial colonization of the nodules (Middleton et al., 2007). Shortly after, a yeast one-hybrid experiment identified *MtERN1*, *MtERN2*, and *MtERN3* as transcription factors of the group V of the ERF subfamily that directly bind to a *cis*-element, the NF-box, present in the *MtENOD11* promoter (Andriankaja et al., 2007). Binding of MtERN1 and MtERN2 to the NF-box leads to activation of gene expression of *MtENOD11* (Andriankaja et al., 2007). MtERN3 is thought to act as a transcriptional repressor of *MtENOD11* (Andriankaja et al., 2007); however, its function during symbiosis has not been yet genetically characterized (**Figure 1**). *MtERN1* and *MtERN2* showed partially overlappingexpression patterns and partially redundant functions during bacterial infection and nodulecolonization (Cerri et al., 2016). Upregulation of *MtERN1* in root hairs in response to infection by rhizobia or NF application depends on the NF receptor NFP, the CCaMK and the GRAS transcription factors NSP1 and NSP2 (Andriankaja et al., 2007; Middleton et al., 2007). Moreover, MtERN1 interplays with the NSP1/NSP2 complex by binding to a separate *cis*-element in the *MtENOD11* promoter to activate its expression during NF signaling and rhizobial infection (Hirsch et al., 2009; Cerri et al., 2012) (**Figure 1**). In *M. truncatula* and *Phaseolus vulgaris* (common bean), *MtERN1* is also a direct target of NF-Y, a heterotrimeric transcriptional complex controlled by the central regulator NIN (Laloum et al., 2014; Roda et al., 2024). In *L. japonicus*, *LjERN1* was shown to be activated in response to rhizobia by the direct action of the complex formed by CCaMK and CYCLOPS (Cerri et al., 2017). Similarly to that observed in *M. truncatula*, rhizobial infection was abolished in *L. japoncus ern1* mutants (Cerri et al., 2017; Kawaharada et al., 2017). Despite differences in its regulation by upstream transcription factors, ERN1 seems to act as a positive modulator of rhizobial infection and nodule colonization in both determinate and indeterminate nodules. In *L. japonicus*, another member belonging to the group IX of the ERF family, *LjERF1*, was found to be activated very early after rhizobial infection, playing a positive role in nodule formation (El Yahyaoui et al., 2004; Asamizu et al., 2008). *LjERF1* is also induced by both ethylene and JA, and its silencing increased transcript levels of the pathogenesis related gene *LjPR10-1.* Thus, it has been suggested that this transcription factor might be a convergent point for ethylene and JA that contribute to the suppression of defense responses to allow successful rhizobial infection (Asamizu et al., 2008). Since its closest homolog in Arabidopsis, *ERF1*, is a target of EIN3, *LjERF1* might be one of the downstream transcription factors activated by the EIN2/EIN3 module. However, the analysis of this gene in a *skl/ein2* background has not been yet investigated.

Two transcription factors belonging to the ERF subfamily group V have been shown to act at later stages of the root nodule symbiosis in *M. truncatula*, namely Ethylene response Factor required for nodule Differentiation (MtEFD) and MtEFD2 (Vernié et al., 2008; Jardinaud et al., 2022). Unlike *MtERN1* and *MtERN2*, *MtEFD* is not induced by NFs, and only mildly induced by *Sinorhizobium meliloti* at early stages of the symbiotic interaction, however it is highly expressed in young nodules, particularly in the nodule infection zone (Vernié et al., 2008). Plant with a loss of function mutation in *MtEFD1* (*efd-1*) exhibited higher frequency of infection events and hypernodulation, but produced small, non-fixing nodules defective in plant and bacterial cell differentiation (Vernié et al., 2008). High throughput expression analysis of *efd-1* mutants and MtEFD overexpressing roots along with transactivation assays identified the *Response Regulator 4* (*MtRR4*) as a putative target of MtEFD. *MtRR4* encodes a type-A RR involved in the negative control of the cytokinin pathway. It has been hypothesized that the MtEFD-mediated activation of *MtRR4* is required for inhibition of the cytokinin signaling, with the subsequent inhibition of nodule initiation and differentiation (Vernié et al., 2008). More recent experiments showed that expression of MtRR4 in an *efd-1* background reverted the hypernodulation phenotype of the *efd-1* mutant, but it was insufficient to restore nodule differentiation and nitrogen fixation, indicating that other players downstream of MtEFD might be involved at these stages of the root nodule symbiosis (Jardinaud et al., 2022). This report also showed that the *efd-1* mutant exhibits reduced endoreduplication of both plant and bacterial cells, a hallmark of nodule differentiation. RNA-sequencing comparing WT and *efd-1* immature nodules not only confirmed MtEFD dependent upregulation of *MtRR4* but further revealed downregulation of genes encoding Nodule Cystein Rich (NCR) peptides, some of which are required for bacteroid differentiation, and *MtCCS52*, a cell cycle gene controlling endoreduplication of plant cells (Jardinaud et al., 2022) (**Figure 3**). MtEFD and its paralog MtEFD2 exhibit high sequence similarity in the N-terminal region containing the AP2/ERF DNA binding domain but differ in the C-terminal region. Domain swap complementation experiments highlighted the importance of neofunctionalization of the C-terminal region of MtEFD2. Interestingly, *MtEFD2* seems to counteract the negative role of *MtEFD1* on nodule inception (Jardinaud et al., 2022). In addition, MtEFD2 contributes to the upregulation of *MtNF-YA1* and *MtNF-B16*, as well as a *YUCCA* gene involved in auxin biosynthesis and six *SHORT INTERNODES/STYLISH (SHI/STY)* genes involved in the auxin signaling cascade (Jardinaud et al., 2022) (**Figure 3**). Altogether, this evidence suggests that *MtEFD* and *MtEFD2* paralogs seem not only to have undergone distinct neofunctionalization during the evolution of the root nodule symbiosis, but also developed different connections with hormones, with MtEFD1 being linked to cytokinin signaling and response, and MtEFD2 to the activation of genes involved in auxin biosynthesis.

*PLETHORA* (PLTs) genes encode transcription factors of the AP2 subfamily with critical functions in the establishment of the root stem cell niche during embryonic development in Arabidopsis (Aida et al., 2004). *PLTs* genes are transcriptionally activated by auxin and display a gradient mRNA distribution with maxima in the stem cell area of the root, where they promote stem cell identity and maintenance. Low *PLT* mRNA levels in stem cell daughters promote mitotic activity, whereas cell differentiation requires further reduction of *PLT* transcripts (Galinha et al., 2007). Interestingly, *PLT* genes in *M. truncatula* are expressed in the nodule primordia and nodule meristem. *MtPLT1* and *MtPLT2* are detected predominantly in the vasculature-associated nodule meristem domain, whereas *MtPLT3* and *MtPTT4/BABY BOOM* (*MtBBM*) are mainly expressed in the central nodule meristem (Franssen et al., 2015). Knockdown of *MtPLT1*/*MtPTL2*, *MtPLT3*/*MtPLT4* or of all four *PLTs* using an RNAi construct under the control of the early symbiotic *MtENOD12* promoter reduced nodule number and size and altered proper formation and maintenance of the nodule meristem (Franssen et al., 2015) (**Figure 3**). Considering the function of PLTs in the establishment and patterning of the root meristem in Arabidopsis, and the expression pattern and function of PTLs in the formation and maintenance of the nodule meristem, it has been proposed that PLTs might be components recruited from the root meristem developmental programs that legumes incorporated into the program of nodule organogenesis.

Another member of the AP2/ERF family implicated in the control of nodule number is the so-called *Nodule Number control 1* (*NNC1*). *NNC1* was originally identified in soybean and later in common bean and *M. truncatula* as a negative modulator of the formation of both determinate and indeterminate nodules (Wang et al., 2014; Nova-Franco et al., 2015; Traubenik et al., 2025). Expression of *NNC1* is post-transcriptionally regulated by the microRNA172 (miR172). Moreover, it has been evidenced that miR172-mediated endonucleolytic cleavage of *MtNNC1* requires a functional SUPERKILLER complex, which might be implicated in the 3´ to 5´degradation of the 5´cleavage product of *NNC1* (Traubenik et al., 2025). Interestingly, upregulation of miR172 in response to rhizobia in soybean depends on the miR156/S*QUAMOSA promoter‐binding protein‐like 9d* (*GmSPL9d*) module, since binding of GmSPL9d to the miR172 promotor activates its expression (Yan et al., 2013; Yun et al., 2022). GmNNC1 binds to the promoter of the G*mENOD40*, repressing its transcription. Upon inoculation with rhizobia, NIN-mediated increase of miR172 levels promotes endonucleolytic cleavage of the *GmNNC1* transcript, releasing the repression of *GmENOD40*, which is required for the activation of cortical cell division that will form the nodule primordia (Wang et al., 2014). Expression of *ENOD40* in the cortex also seems to be under the control of NNC1 in common bean and *M. truncatula*, since roots silenced in this AP2/ERF member exhibited enhanced expression of *ENOD40* (Nova-Franco et al., 2015; Traubenik et al., 2025). Since NIN is activated by the cytokinin signaling pathway in the cortex, *NNC1* degradation in this cell layer appears to be under the control of this hormone (**Figure 1**). Interestingly, *MtNNC1* also has been shown to participate in the control of rhizobial infection, since roots silenced in *MtNNC1* exhibited higher density of infection events that do not necessarily progress to the cortical cells (Traubenik et al., 2025); however, its link to hormones during infection remains unexplored. At later stages of the root nodule symbiosis, *GmNNC1* participates in the host-controlled AON by repressing the expression of two CLE peptides encoding genes, *GmRIC1* and *GmRIC2* (Wang et al., 2019). Transcriptional activation of *GmRIC1* and *GmRIC2* is a hallmark for activation of AON signaling in the shoot. Thus, overexpression of the miR172 resistant form of *GmNNC1* blocks AON signaling, leading to uncontrolled nodule formation. In wild type plants, infection by rhizobia induces GmNIN-mediated expression of miR172, removing *GmNNC1* repression of *GmRIC1* and *GmRIC2.* In turn, GmNIN also physically interacts with GmNNC1, releasing transcriptional repression of G*mRIC1, GmRIC2*, and miR172. Thus, the GmNINa-miR172c-GmNNC1 network functions as a hub that coordinately regulates AON signaling, providing a balance between nodule formation and AON in soybean (Wang et al., 2019).

Two soybean genes belonging to the ERF subfamily, designated as *GmENS1* and *GmENS2* (*Ethylene-responsive transcription factors required for Nodule Senescence*), have been implicated in nodule senescence (Xiao et al., 2024). Transcripts of these genes are expressed at high levels in aged or ethylene treated nodules. Overexpression of *GmENS1* and *GmENS2* accelerates nodule senescence, whereas knockout or knockdown of both genes delay senescence of the nodules, which exhibit elevated nitrogenase activity as compared with wild type plants. Moreover, GmENS1 and GmENS2 directly bind to the promoter and activate the expression of three genes encoding NAC transcription factors, GmNAC039, GmNAC018, and GmNAC030, promoting nodule senescence (Xiao et al., 2024) (**Figure 3**). Recently, another member of the AP2/ERF family in soybean, GMERF13s, has been shown to play a key role in nodulation under salt stress conditions. Nodule formation inhibition caused by salt stress is attenuated in *GmERF13s* loss of function mutants and enhanced in roots overexpressing *GmERF13s*. In addition, GmERF13s can interact with Lateral Organ Boundaries Domain GmLBD16a, interfering with the GmLBD16 mediated activation of the gene *Expansin 17* (*GmEXP17*), which promotes nodule formation (Zhu et al., 2025).

All this evidence indicates that AP2/ERF transcription factors constitute a diverse family with multiple functions in the root nodule symbiosis, exerting positive and negative effects on infection and nodule organogenesis under normal or stress conditions, mediating nodule meristem establishment and promoting endoreduplication of bacterial and plant cells during nodule differentiation. Their expression is tightly modulated in the specific cell types engaged in the root nodule symbiosis interplaying with hormonal balance, mainly cytokinin, auxin and ethylene. At later stages, members of this family also participate in the intricate mechanisms of systemic control of nodule number, as well as in nodule senescence by activation of the senescence related *NAC* genes.

### Nuclear factors Y play key function in infection, nodule development and strain specificity

Nuclear Factor Ys (NF-Ys) are heterotrimeric transcription factors composed of three subunits, NF-YA, NF-YB and NF-YC, which binds with high specificity to the CCAAT box present in many eukaryotic promoters. The NF-YA subunit contains a DNA binding domain, whereas NF-YB and NF-YC contain histone-fold motifs (HFMs) that participate in the dimerization of these two subunits. Subsequent binding of the NF-YA subunit to the NF-YB/NF-YC heterodimer forms a functional heterotrimeric complex that binds to the DNA (Wu et al., 2025). In plants, each subunit is encoded by multiple genes that have functionally diversified, participating in multiple developmental processes and adaptive responses to the environment (Petroni et al., 2012; Zanetti et al., 2017). In the context of the root nodule symbiosis, individual members of the three subunits are transcriptionally activated by NIN as a function of increased cytokinin concentration and/or signaling (Soyano et al., 2013; Liu et al., 2019; Schiessl et al., 2019). These NF-Y members play key functions during infection by rhizobia, nodule formation and nodule differentiation establishing regulatory circuits with other transcriptional regulators that activate auxin signaling in different legume species (Schiessl et al., 2019; Soyano et al., 2019; Shrestha et al., 2021).

In *M. truncatula*, *MtNF-YA1* (formerly *MtHAP2-1*) plays an essential function in the formation and maintenance of the nodule meristem (Combier et al., 2006), the place of localized auxin maxima during nodule development (Xiao et al., 2025). Silencing or mutation of this gene produces undifferentiated nodules that lack a persistent meristem (Combier et al., 2006), as well as impaired upregulation of genes encoding Auxin Response Factor (ARF) and SHI/STY transcription factors linked to auxin signaling and response during nodule development, as will be described in the following sections (Kirolinko et al., 2021; Shrestha et al., 2021). Expression of *MtNF-YA1* is spatially restricted to the nodule meristem at early stages of nodule development by a post-transcriptional repression mechanism mediated by the microRNA169 (miR169) (Combier et al., 2006; Laporte et al., 2014). More recently, it was shown that the *MtENOD40* RNA functions as a mimicry that sequestrates miR169 releasing repression of *MtNF-YA1*, and promoting nodule formation (Wijsman et al., 2025) (**Figure 1**). Intriguingly, in soybean, the miR169/*GmNF-YAc* module controls the expression of *GmENOD40* during nodulation in response to nitrogen availability (Xu et al., 2021). Thus, the miR169/NF-YA module seems to control expression of *ENOD40* and the latter seems to release miR169-mediated post-transcriptional repression of *NF-YA* expression, maintaining high levels of the *NF-YA* transcript. These results suggest an intricated positive feed-forward loop that maintains high levels of both *NF-YA* and *ENOD40* during infection by rhizobia and nodule initiation, however, whether both the transcriptional and post-transcriptional mechanisms operate simultaneously in determinate and/or indeterminate types of nodules remain to be investigated. At later stages of nodule development, alternative splicing of *MtNF-YA1* produces a peptide encoded by an upstream open reading frame (uORF1), which binds to both *MtNF-YA1* transcript variants promoting its degradation in the nodule infection zone, thereby restricting *MtNF-YA1* expression to the meristem of mature nodules (Combier et al., 2008).

*MtNF-YA1* has been also linked to rhizobia infection. *Mtnf-ya1* mutants showed delayed infection and thicker and branched ITs typically observed when their growth is arrested (Laporte et al., 2014). Another member of the NF-YA family, *MtNF-YA2*, acts redundantly with *MtNF-YA1* in the control of rhizobial infection and the activation of the symbiotic genes *MtERN1* and *MtENOD11* (Laloum et al., 2014). Both MtNF-YA1 and MtNF-YA2 subunits interact with the MtNF-YC1 and MtNF-YC2 subunits, as well as with MtNF-YB16 (Baudin et al., 2015). Knockdown of *MtNF-YC1* and *MtNF-YC2* reduced nodule formation and affected nodule differentiation (Baudin et al., 2015). Moreover, MtNF-YC2 and MtNF-YB16 also binds to the *MtERN1* promoter, suggesting that MtNF-YA1/A2, MtNF-YB16 and MtNF-YC1/C2 form a functional heterotrimeric complex during symbiosis (Baudin et al., 2015) (**Figure 1**).

In *L. japonicus*, *LjNF-YA1* and *LjNF-YB1* are direct transcriptional targets of NIN as a function of cytokinin signaling in the root cortex (Soyano et al., 2013). *LjNF-YA1* and *LjNF-YB1* are co-expressed in the dividing cortical cells of root nodule primordia and interact with each other to function together in nodule development. Knockdown of *LjNF-YA1* severely reduced nodule formation, suggesting that cortical cell divisions in nodule organogenesis require *LjNF-YA1.* This was further supported by the fact that constitutive expression of *LjNF-YA1* enhanced cell divisions in the root, whereas its co-expression with LjNF-YB1 exaggerated these cell divisions (Soyano et al., 2013). LjNF-YA1 has been shown to control the expression of seven members of *the SHI/STY* transcription factor gene family, which in turn regulate *YUCCA* genes involved in the biosynthesis of auxin (Shrestha et al., 2021). In addition, it was recently shown that the soybean GmNF-YAc transcription factors directly activate the root stem-cell regulator WUSCHEL-RELATED HOMEOBOX gene (WOX5) in the cortex, initiating the cortical cell divisions that will form the nodule primordia (Li et al., 2026) (**Figure 1**). Surprisingly, in contrast with that observed in *M. truncatula nf-ya1* mutants (Laporte et al., 2014), knockdown of *LjNF-YA1* did not affect the initiation or progression of ITs (Soyano et al., 2013).

In common bean, individual members of the NF-YA, NF-YB and NF-YC families have been implicated not only in the control of rhizobial infection and nodule organogenesis, but also in the preference of the plant by rhizobia strains that are more efficient in nodulation. Expression of *PvNF-YC1*, *PvNF-YA1* and its close paralog *PvNF-YA9* are activated in common bean by strains that are highly efficient in nodule formation (Zanetti et al., 2010; Mazziotta et al., 2013; Ripodas et al., 2019). Knockdown of *PvNF-YC1* or *PvNF-YA1/A9* arrests nodule formation by either low or high efficient strains, presumably by impairing activation of cell division mediated by G2/M cell cycle genes (e.g. *CDC2*, *CyclinB* and *CDC25*) (Zanetti et al., 2010). Moreover, PvNF-YC1 directly targets a gene encoding a member of the P4 cyclin family, which is required for nodule formation and infection, providing a direct connection between NF-Ys and a cell cycle gene (Roda et al., 2024). On the other hand, overexpression of PvNF-YC1 or PvNF-YA1 was sufficient to enhance nodule formation by low efficient strains and to alter nodule occupancy by high and low efficient rhizobia strains (Zanetti et al., 2010). PvNF-YC1 was shown to physically interact with the PvNF-YA1 and PvNF-YB7 subunits (Baudin et al., 2015) Knockdown of *PvNF-YB7* affected nodule formation exclusively in the interaction of common bean with a highly efficient strain, restraining also nodule occupancy by this strain in co-inoculation experiments (Ripodas et al., 2019). Thus, PvNF-YA1, PvNF-YB7 and PvNF-YC1 form a heterotrimer that controls not only the establishment of functional nodules, but also the selection of the bacterial strain that will occupy the nodules. PvNF-YC1 also physically interacts with a GRAS transcription factor named SCL13 Involved in Nodulation (SIN1) (Battaglia et al., 2014). Interestingly, SIN1 controls the expression of cell cycle genes and *PvNF-YA1* in nodule and lateral root formation, providing another connection between both developmental programs (Battaglia et al., 2014).

NF-Ys transcription factors affect each step of the root nodule symbiosis, from pre-infection stages to nodule differentiation. Expression of NF-Y subunits in response to rhizobia seems to be a function of cytokinin signaling and the central regulator NIN. In turn, NF-Ys control the expression of genes required for rhizobial infection, cell cycle progression and auxin biosynthesis to mediate the formation of functional fixing nodules. Further research will help to unveil the connection between NF-Y transcription factors and the role of hormone-mediated signaling in cell-cycle control and rhizobia infection in other legume species.

### Auxin response factors are required for the auxin-mediated response to rhizobial infection and nodule organogenesis

Auxin plays an important role in root growth and development by inhibiting primary root growth and promoting lateral root emergence (Benková et al., 2003; Ivanchenko et al., 2008; Péret et al., 2009; De Smet, 2012), as well as in the nitrogen-fixing symbiosis between legumes and rhizobia (Mathesius, 2008; Kohlen et al., 2018). Auxin responses are mediated by a family of transcription factors known as Auxin Response Factors (ARFs) and members of the Aux/IAA protein family. At low concentrations of auxin, some ARFs form heterodimers with Aux/IAA, which recruit the co-repressor TOPLESS at the promoters of Auxin-Responsive Genes (ARGs). At high auxin concentrations, these phytohormones are sensed by the Transport Inhibitor Response 1/Auxin-Signaling F-box1-3 (TIR1/AFB1-3) receptor, mediating Aux/IAA protein ubiquitination and subsequent degradation, releasing ARF repression and promoting ARF-mediated transcriptional activation of ARGs (Tiwari et al., 2003; Chandler, 2016). ARFs have been classified either as activators or repressors, and mediate transcriptional regulation through their binding to Auxin Response Elements (AuxREs) present in the promoter regions of ARGs. ARFs recognize AuxREs by their first four bases, TGTC, and bind to this *cis*-regulatory element through their DNA binding domain to modulate gene expression (Ulmasov et al., 1999; Li et al., 2016; Weijers and Wagner, 2016). In *A. thaliana*, ARF genes are divided into three main clades: clade A, which are activators, clade B, which are repressors, and clade C, which may act as both activators and repressors (Finet et al., 2013). However, it has been recently shown that ARFs transcriptional properties are not simply dependent on their clade of precedence but are also modulated by the AuxREs pairs they bind to, adding another layer of complexity to auxin-responsive regulation (Martin-Arevalillo et al., 2025).

Several members of the ARF family have been involved in the root nodule symbiosis across different legume species, with most of them having their expression post-transcriptionally regulated by miRNAs. The role of ARFs varies between different members of the family, acting at different stages of symbiosis. ARF6 and ARF8 are targets of miR167. High levels of the miR167 were found in nodules of *L. japonicus* (De Luis et al., 2012) and soybean, where upregulation of miR167 promotes post-transcriptional cleavage of its target transcripts *GmARF6*, *GmARF8a* and *GmARF8b* (Wang et al., 2015). The post-transcriptional repression of *GmARF8a* and *GmARF8b* by miR167 was demonstrated to be crucial for soybean nodule development, enhancing nodule number during symbiosis (Wang et al., 2015). Other studies revealed that levels of miR160 increased during nodulation, and overexpression of this miRNA reduced nodule number by repressing *GmARF10, GmARF16* and *GmARF17* transcript levels (Turner et al., 2013; Nizampatnam et al., 2015). The work of Turner et al. (2013) showed that the auxin hypersensitivity caused by overexpression of miR160 did not affect the epidermal response to rhizobia but impaired the subsequent development of nodule primordia. Similarly, in *M. truncatula*, overexpression of miR160 reduced nodule number upon rhizobial inoculation, possibly by downregulation of *MtARF10, MtARF16* and *MtARF17* (Bustos-Sanmamed et al., 2013). In this legume, miR160 levels increased at early stages of the interaction with *S. meliloti*, but this early induction is blocked in the non-nodulating mutants *dmi1* and *nfp*, suggesting that the action of miR160 depends on the NF signaling pathway (Bustos-Sanmamed et al., 2013). Promoter-reporter expression assays showed that *MtARF16* is activated in infected hairs, around infection foci and along the ITs upon inoculation with *S. meliloti* (Breakspear et al., 2014). Moreover, *arf16a* mutants showed a lower number of infection events, indicating that *MtARF16* is likely to be required for initiation of infections in indeterminate nodules (Breakspear et al., 2014) (**Figure 1**), which contrasts with that observed in soybean determinate nodules (Turner et al., 2013).

Expression of the *ARF2*, *ARF3* and *ARF4* members is modulated by the action of the miR390/*Trans-Acting Small Interfering RNA* 3 (*TAS3*) pathway during lateral root development in *A. thaliana* (Marin et al., 2010). In *M. truncatula*, it has been observed that knockdown of *MtARF2/3/4a/4b* or mutation of *MtARF4a* impaired nodule development and reduced initiation and progression of infection events in response to rhizobia (Hobecker et al., 2017; Kirolinko et al., 2021). Interestingly, these plants also exhibited shorter primary and lateral roots but increased lateral root density, suggesting that MtARF2/3/4 could have been recruited from the lateral root development program. Silencing of *MtARF2/3/4a/4b* also impaired induction of the early symbiotic gene *MtNSP2* (Kirolinko et al., 2021). This is consistent with previous results indicating that activation of the miR390/*TAS3* module by overexpression of miR390, which decreased levels of *MtARF2/3/4*, reduced nodule number and infection, as well as the induction of *MtNSP1* and *MtNSP2* (Hobecker et al., 2017) (**Figure 2**). More recently, chromatin immunoprecipitation (ChIP) assays demonstrated that MtARF2 directly binds to AuxREs present in the promoters of *MtNSP2, MtARF4a*, and the member of the Lateral Organ Boundaries Domain (LBD) family *MtLBD17/29a* in rhizobia inoculated roots (Kirolinko et al., 2024). These results reveal a crosstalk between components the lateral root developmental program and the NF signaling pathway that mediate nodule formation in response to increased auxin signaling.

ARF5/MONOPTEROS (MP) has been shown to be involved in cotyledon, root pole and vasculature development (Hardtke and Berleth, 1998), as well as in LRs emergence and elongation (De Smet et al., 2010). Its role in nitrogen-fixing symbiosis is not known yet; however, a transcriptome study in soybean indicated that *GmARF5* is induced in emerging and mature nodules (Smita et al., 2020). In addition, an analysis of *M. truncatula* expression data showed that transcript levels of *MtARF5* increased in the shoot and decreased in the roots at early time points after inoculation with rhizobia (Shen et al., 2015). Considering that in *A. thaliana* ARF5/MP has been shown to recruit chromatin remodeling proteins to enhance the DNA accessibility of other transcriptional activators upon auxin sensing during leaf and flower development (Wu et al., 2015; Chandler, 2016), it would be interesting to investigate whether ARF5 or other members of the ARF family have a similar role promoting chromatin accessibility during infection and nodule development in legumes. On the other hand, even though *ARF7* and *ARF19* have shown to play a central role in lateral root formation through auxin-induced modulation of *LBD16* in Arabidopsis (Okushima et al., 2007), RNA-seq data reported by Schiessl et al. (2019) indicated that none of these ARFs are induced during lateral root formation or nodule development in *M. truncatula* roots. However, another expression analysis revealed that *MtARF7* levels increased in the shoot upon inoculation with *S. meliloti* (Shen et al., 2015), suggesting that *MtARF7* might play a role in long distance signaling during nodulation. Further analysis of the ARF family members, their direct targets and homo-and heterodimerization of members of this family are required to better understand the role of ARFs in different tissues during nitrogen-fixing symbiosis.

### Lateral organ boundaries domain transcription factors: from lateral root development to root nodule symbiosis

The Lateral Organ Boundaries Domain (LBD) proteins constitute a family of plant-specifictranscription factors defined by the presence of a conserved domain known as LOB (Lateral OrganBoundaries) that modulate multiple developmental processes, including root development and noduleorganogenesis (Schiessl et al., 2019; Soyano et al., 2019, Soyano et al., 2021; Kirolinko et al., 2024; Schiessl and Jhu, 2025). LBD proteins are composed of a relatively conserved N-terminal region and a variable C-terminal region. The N-terminal region contains the LOB domain, which mediates DNA binding and protein dimerization, whereas the C-terminal region confers transcriptional activation or repression functions (Zhang et al., 2020). According to their structural features, LBD proteins are grouped into two main classes. Class I members contain coiled-coil–forming regions and are further subdivided into four subclasses (IA, IB, IC, and IE). Class II proteins lack a complete leucine-zipper motif and are divided into two subclasses, IIA and IIB (Shuai et al., 2002). *AtLOB* was the first member of the LBD family identified in Arabidopsis. Loss of function mutants of *AtLOB* exhibit organ fusions (Husbands et al., 2007) (Shuai et al., 2002). Subsequent studies demonstrated that *AtLBD29*, *AtLBD18*, and *AtLBD16* are modulated by auxin signaling to mediate developmental programs in roots, including development of lateral and adventitious roots (Okushima et al., 2007; Soyano et al., 2008; Lee et al., 2009, Lee et al., 2015; Omary et al., 2022). In the past years, LBD transcription factors have emerged as central developmental mediators of root nodule organogenesis, acting downstream of cytokinin and symbiotic signals to promote cortical cell division and organ formation (Schiessl et al., 2019; Soyano et al., 2019; Kirolinko et al., 2024). In *Lotus japonicus*, the *LjASL18/LjLBD16a* gene is required for both lateral root and nodule formation. During symbiosis, *LjASL18/LjLBD16a* is directly activated by the central regulator NIN, positioning LBD transcription factors at a convergent point between cytokinin signaling and symbiotic transcriptional control (Soyano et al., 2019). An intronic *NIN* binding site is conserved in *ASL18/LBD16* orthologs of legumes but not in orthologs of non-leguminous species, providing an evolutionary clue that suggests how a gene originally involved in lateral root development could have been recruited for symbiotic nodulation. In *M. truncatula*, transcriptomic analyses revealed that *MtLBD16*, the ortholog of *LjASL18/LjLBD16a*, is induced at very early stages of lateral root and nodule development (Schiessl et al., 2019). *MtLBD16* is also required for proper formation of both lateral root and nodule primordia. During nodulation, loss of function mutation of *MtLBD16* leads to impaired or disorganized cortical cell divisions, underscoring its central role in orchestrating nodule initiation (Schiessl et al., 2019). Activation of *MtLBD16* during nodulation is also a function of the MtCRE1 receptor and NIN. Thus, unlike the canonical auxin-dependent regulation observed during lateral root formation, activation of LBD16 during nodulation relies predominantly on cytokinin signaling and NIN activity (Schiessl et al., 2019; Soyano et al., 2019). Interestingly, *MtLBD16* regulation by NIN is also dependent on the SCR-SHR module, and expression of SCR depends on *MtLBD16* (Dong et al., 2021) (**Figure 2**). These regulatory hubs illustrate how conserved developmental regulators have been integrated into symbiosis-specific signaling pathways, allowing nodule organogenesis to be uncoupled from the classical auxin-dependent regulatory mechanisms.

More recently, a transcriptomic study showed that the induction of *MtLBD17/29a* and *MtLBD33* during early symbiotic stages requires the activation of the miR390/*TAS3*/*ARFs* module (Kirolinko et al., 2024). As mentioned in the previous section, ChIP-PCR experiments verified that *MtLBD17/29a* is a direct target of MtARF2. The binding of MtARF2 to an AuxRE present in the *MtLBD17/29a* promoter activates its transcription in rhizobia inoculated roots (Kirolinko et al., 2024). Upregulation of *MtLBD17/29a* is also NIN dependent (**Figure 1** and **2**), indicating that NIN-mediated activation might be a common feature of *MtLBD16* and *MtLBD17/29a*. However, the spatial expression patterns of *MtLBD16* and *MtLBD17/29a* do not fully overlap in inoculated roots (Schiessl et al., 2019), suggesting that they might function in different cell types during nodule formation. Either knockdown or overexpression of *MtLBD17/29a* leads to a reduction in nodule number and development, and reduced frequency of infection events. Alteration of *MtLBD17/29a* mRNA levels also impaired the induction of the symbiotic genes *NSP1* and *NSP2*, linking this LBD transcription factor with the NF signaling pathway (**Figure 1**). Since silencing and overexpression of this LBD member produced similar phenotypes, it has been suggested that MtLBD17/29a could form transcriptional heterocomplexes with additional transcription factors during symbiosis (Kirolinko et al., 2024). However, this speculation remains to be investigated. On the other hand, knockdown of *MtLBD17/29a* reduced the length of primary and lateral roots and enhanced lateral root formation, whereas overexpression of MtLBD17/29a produced the opposite phenotype (Kirolinko et al., 2024) (**Figure 2**). Thus, MtLBD17/29a represents another LBD member that might have been recruited from root developmental programs to play a function in the root nodule symbiosis.

Together, these findings establish a framework in which LBD transcription factors act as key regulators of developmental programs that initiate postembryonic lateral organs in the root. Comparative and evolutionary studies support the hypothesis that root nodule symbiosis evolved through the recruitment and modification of ancestral developmental programs originally involved in lateral organ formation (Soyano et al., 2021; Schiessl and Jhu, 2025). In this context, LBD transcription factors exemplify this evolutionary process, as their incorporation into nodulation-specific gene regulatory networks provides a mechanistic explanation for the parallels between lateral root and nodule organogenesis and highlights the evolutionary plasticity of plant transcriptional circuits.

### Short internodes/stylish transcriptional activators modulate auxin homeostasis during nodule formation

The SHI/STY family consists of functionally redundant members that modulate the development of diverse plant organs (Kuusk et al., 2006). Members of this family contain two highly conserved regions, a RING-like zinc finger domain and a C-terminal IGGH domain that is unique to the SHI/STY family, as well as one or two Gln-rich regions that suggest that they might function as transcriptional activators. In Arabidopsis, mutations in *AtSTY1* and *AtSTY2* genes altered auxin homeostasis, affecting leaf, flower and lateral root development (Smith and Fedoroff, 1995; Sohlberg et al., 2006; Baylis et al., 2013). Moreover, *AtSTY1* and possibly other related members of this family are transcriptional activators of genes involved in auxin biosynthesis, including the flavin monooxygenase-encoding gene *YUCCA4* (Sohlberg et al., 2006; Eklund et al., 2010; Baylis et al., 2013).

In legumes, *SHI/STY* genes have been shown to play important functions in auxin homeostasis during nodule formation. In *L. japonicus*, LjNF-YA1 directly binds to the promoters of *LjSTY1*, *LjSTY2*, and *LjSTY3* genes, activating their expression in the nodule vascular bundles (Hossain et al., 2016) (**Figure 1**). Promoter:reporter assays showed that eight of the nine *LjSTY* genes were expressed in lateral root primordia and the central vasculature and apex of emerged lateral roots, as well as in actively dividing cortical cells of the nodule primordia (Shrestha et al., 2021). Further genetic characterization of *SHI/STY* genes revealed that single *sty* mutants show weak symbiotic phenotypes, whereas a *sty1–2 sty2–1 sty3–9* triple mutant exhibited fewer nodules and ITs, suggesting that these genes play redundant function during the root nodule symbiosis. The function of *SYT3* was further confirmed by the expression of a negative dominant *STY3::SRDX* chimera, which blocked nodule formation and arrested the initiation and progression of infection events (Shrestha et al., 2021).

In *M. truncatula*, upregulation of members of the *SHI/STY* and *YUCCA* families during lateral root or nodule formation depends on MtLBD16 (Schiessl et al., 2019) (**Figure 2**). During nodule formation, expression of *SHI/STY* and *YUCCA* genes also depends on the cytokinin receptor MtCRE1 and the central regulator NIN. In particular, *MtSTY2* was found to be expressed in the lateral root primordia and in the apex of the main roots, whereas inoculation with rhizobia activates *MtSTY2* expression in dividing cells of the inner cortex that were reached by ITs, and in most dividing cells of the nodule primordia and emerged nodules (Shrestha et al., 2021) (**Figure 1** and **2**). This expression pattern coincides with that of *MtLBD16* during lateral root and nodule development (Schiessl et al., 2019). In mature nodules, *MtSTY2* expression is restricted to the uninfected cells of the vascular bundles and the meristematic zone of the nodule (Shrestha et al., 2021), which is reminiscent of the expression observed for *MtARF4* and *MtLBD17/29* transcription factors (Kirolinko et al., 2021, Kirolinko et al., 2024). Upregulation of *MtLBD17/29* in response to rhizobia is also *NIN* dependent (Kirolinko et al., 2024). Expression of *MtNF-YA1* depends on *MtLBD17/29a*, whereas expression of *MtARF4a* requires *MtNF-YA1* (Kirolinko et al., 2021). Thus, a possible scenario is that NIN and MtLBD16 promote auxin biosynthesis via activation of *SHI/STY* and *YUCCA* genes in dividing cortical cells beneath the infection site, and in the meristem and uninfected cells of mature nodules. This localized auxin maximum interplays with MtNF-YA1 to promote activation of *MtARF4*, and further activation of *MtLBD17/29a*-dependent expression of *MtNF-YA1* forms a positive loop that mediates rhizobial infection and the development of indeterminate nodules.

### Other transcription factors linked to hormones: WUSCHEL-related homeobox and nodule root

Other genes encoding transcription factors linked to hormones with key roles in the root nodule symbiosis include the WOX and NOOT families. *WOX* genes encode homeodomain-containing transcription factors that participate in the balance between cell division and differentiation in the root apical meristem (Sarkar et al., 2007). In legumes forming indeterminate nodules, including *M. truncatula* and pea, WOX5 has been shown to be upregulated in the nodule primordia at early time points after infection by rhizobia, as well as in the vascular bundles of the meristem of mature nodules (**Figure 1**). *WOX5* is also upregulated upon auxin exogenous treatment (Osipova et al., 2012). The function of this family of transcription factors in the root nodule symbiosis has been recently elucidated in soybean, showing that a *wox5abc* triple mutant have reduced nodule number and attenuated nitrogen fixation (Li et al., 2026). This study also showed that *GmWOX5* is a direct target of the symbiotic regulator GmNF-YAc. On the other hand, *NOOT* genes belong to the NPR1 family of transcriptional coactivators, which contain a BTB/POZ domain and ankyrin repeats. The *Nodule root* 1 gene in *M. truncatula* (*MtNOOT1*), the *NOOT-BOP-COCH-LIKE1* gene in *L. japonicus* (*LjNBCL1*) and the *COCHELATA* gene in pea (*PsCOCH*) have been implicated in the maintenance of the identity of the nodule meristem, since loss of function mutants of these genes developed ectopic roots that arise from the nodule vascular meristem (Couzigou et al., 2012; Magne et al., 2018a, Magne et al., 2018b) (**Figure 3**). A paralog of *MtNOOT1*, designated *MtNOOT2*, was also identified (Magne et al., 2018b). Expression of *MtNOOT1* and *MtNOOT2* does not overlap, since *MtNOOT1* is expressed in the nodule vascular meristem, whereas *MtNOOT2* is expressed in the nodule central meristem. A *Mtnoot2* single mutant does not exhibit a symbiotic phenotype, however *Mtnoot1 noot2* double mutants completely losses nodule meristem identity and develop nodule-like structures that were unable to host rhizobia, indicating that both genes are required for the maintenance of nodule meristem identity and successful colonization of the nodule cells (Magne et al., 2018a). Transcriptomic approaches have shown that expression of auxin and cytokinin signaling genes are disrupted in *Mtnoot Mtnoot2* double mutants or in *MtNOOT/MtNOOT2* overexpression lines (Lee et al., 2024). More recently, *PsCOCH* has been linked to the spatial regulation of auxin and cytokinin during nodule organogenesis. *Pscoch* mutants showed abnormal nodules with elevated levels of cytokinin, which is accompanied by higher expression levels of the cytokinin reporter gene *TCSn::GUS* in the nodule apex, vasculature and ectopic root-like tissues; as well as reduced auxin levels and an altered expression pattern of the auxin response reporter DR5:GUS (Velandia et al., 2025). However, considering the role of auxin and cytokinin in nodule organogenesis and development, it remains unclear whether expression of *NOOT* genes is modulated by auxin and cytokinin signaling pathways involved in the root nodule symbiosis. In addition, since NOOT protein seem to act as transcriptional coregulators, it will be important to identify the transcription factors that interact with NOOTs during nodule formation.

## Conclusion and perspectives

As described in detail in the previous section, hormone-modulated transcription factors playessential roles orchestrating changes in gene expression at each step of the root nodule symbiosis,including pre-infection, formation and progression of ITs, activation of cortical cell division toform nodule primordia, as well as nodule differentiation and senescence. Auxins and cytokinins have positive effects on nodule development, a different scenario than lateral root development, where both types of hormones seem to have antagonistic roles (Bensmihen, 2015). Auxin maxima are linked to both bacterial infection and nodule organogenesis (Breakspear et al., 2014; Xiao et al., 2025). Auxin levels in response to rhizobia are a function of the NF-Y and SHI/STY transcription factors (Hossain et al., 2016), whereas auxin signaling and response are mediated by the ARF transcription factors during infection and nodule organogenesis (Bustos-Sanmamed et al., 2013; Turner et al., 2013; Breakspear et al., 2014; Wang et al., 2015; Kirolinko et al., 2021). Cytokinin signaling act downstream of NF perception and the CSSP (Gonzalez-Rizzo et al., 2006; Murray et al., 2007; Tirichine et al., 2007; Plet et al., 2011; Reid et al., 2017) to regulate *NIN* expression in the cortical cells through a remote *cis*-regulatory element present in the *NIN* promoter (Liu et al., 2019), triggering NIN-mediated activation of NF-Y and LBD transcription factors and promoting the cortical cell divisions required to form the nodule primordium (Schiessl et al., 2019; Soyano et al., 2019; Kirolinko et al., 2024). The role of cytokinin in infection by rhizobia remains controversial, since mutants in the cytokinin receptor in *M. truncatula* and *L. japonicus* have shown contrasting infection phenotypes (Gonzalez-Rizzo et al., 2006; Murray et al., 2007). GA modulates positively nodule organogenesis in the cortex but negatively rhizobia infection in the epidermis, acting mainly through the regulation of DELLA proteins and interplaying with cytokinins and NIN (Fonouni-Farde et al., 2017; Gao et al., 2023). Brassinosteroid signaling seems to negatively affect rhizobial infection and nodulation by inactivating NSP1/NSP2 heterocomplex (Chen et al., 2023). Nodule differentiation is also controlled by auxin/cytokinin levels. PLT, WOX and NF-Y are modulated by auxin and cytokinin to establish and maintain the identity of the meristem of indeterminate nodules (Osipova et al., 2012; Franssen et al., 2015; Li et al., 2026), which is thought to be recruited from the program of root meristem identity. Differentiation of plant and bacterial cells in the infection and fixation zone is controlled by EFD transcription factor, impairing cytokinin signaling via the RR4 transcription factor and activating auxin biosynthetic genes (Jardinaud et al., 2022). Further research will help to understand how these hormones interplay with transcription factors belonging to other families, i.e., NOOT, to control nodule meristem identity.

Our understanding of the connection between transcription factors and hormones has significantly progressed over the last years, but many questions remain unanswered. Transcription factors function within a complex regulatory landscape to control gene expression (Strader et al., 2022). In this context, future investigation will help to elucidate whether different transcription factors implicated in root, lateral root and nodule development physically interact with each other to form distinct transcriptional complexes that modulate downstream genes required for each of these developmental processes. Some of the transcription factors orchestrating the root nodule symbiosis have been recruited from other developmental programs including root development, lateral root formation and the response to nitrate, and seem to have undergone neofunctionalization in legumes through the acquisition of *cis*-regulatory elements that activate its expression in response to changes in the hormonal balance caused by rhizobia infection. These *cis*-regulatory elements have been identified in NIN, SCR, SHR, NF-Y and LBD genes (Liu et al., 2019; Soyano et al., 2019; Dong et al., 2021), however, it remains to be elucidated whether other, yet unidentified, *cis*-regulatory elements are also the cause of neofunctionalization of additional hormone-modulated transcription factors that have been recruited from other developmental programs to play roles in the root nodule symbiosis. Advances in computational approaches combined with ChIP-seq or DNA Affinity Purification (DAP)-seq experiments will certainly contribute to the identification and experimental validation of the functionality of such *cis*-regulatory elements controlling the expression of hormone-modulated transcription factors and downstream regulated genes during different steps of the root nodule symbiosis. Importantly, to act as repressors or activators, transcription factors must recognize and bind these *cis*-regulatory elements. The accessibility of transcription factors to these *cis*-regulatory elements is influenced by the chromatin conformation state, which relays on epigenetic marks and chromatin remodelers (Zanetti et al., 2024). Epigenetic marks, including DNA methylation and histone posttranslational modification, and chromatin accessibility has been investigated during the root nodule symbiosis (Pecrix et al., 2018; Knaack et al., 2022; Pecrix et al., 2022). The study from Pecrix et al. (2018) revealed that repressive histone marks such as the trimethylation of histone 3 at lysine 27 (H3K27me3) within symbiotic island (genomic regions containing high density of symbiotic genes) are more abundant in roots than in nodules, suggesting that this repressive mark is removed during nodule formation. The study of Knaack et al. (2022) combined transcriptomic and ATAC-seq data to predict *cis*-regulatory elements and transcription factors that significantly contribute to transcriptional changes associated with symbiosis, identifying ethylene (ERF1, EIN3), auxin (SHY2) and abscisic acid (ABI4 and ABI5) related transcription factors as important regulators of gene expression during nodulation. A subsequent phylogenomic and phylotranscriptomic analysis revealed that conserved non-coding elements specific of the NFC are enriched in the epigenetic active mark H3K9ac in nodule and root tissues, which overlap with ATAC-seq peaks, suggesting that those elements present in open chromatin regions might be involved in the transcriptional activation of genes related to the root nodule symbiosis (Zhang et al., 2024). However, how chromatin remodelers and enzymes involved in the deposition or removal of epigenetic marks interplay with hormone-modulated transcription factors during nodulation remains to be investigated. In this regard, the NF-Y and ARF families have been proposed to act as pioneer transcription factors that bind to *cis*-regulatory elements present in genomic regions with a condensed chromatin conformation state (Gurtner et al., 2008; Wu et al., 2015; Chandler, 2016; Tang et al., 2017). Pioneer transcription factors recruit chromatin remodelers and DNA- and histone-modifying enzymes that change chromatin accessibility, exposing additional *cis*-regulatory elements bound by other transcription factors. Advancement in epigenomic approaches, including ChIP-seq and chromatin accessibility experiments, will be critical to identify hormone-modulated transcription factors involved in root and nodule development that could function as pioneer transcription factors, and whether they are able to facilitate the binding of additional transcription factors to promote dynamic transcriptional reprogramming at different developmental stages.
